# A Rare Massive Seromucinous Cystadenoma of the Ovary in a 26‐Year‐Old Female Persisting for 9 Years: A Case Report

**DOI:** 10.1155/crog/5887168

**Published:** 2026-04-09

**Authors:** Marah Kakeh, Mohammad Alhalabi, Wael Wazan, Rasha Abbassi, Abeer Kanjawi, Haitham Abbassi

**Affiliations:** ^1^ Department of Obstetrics and Gynecology, University of Damascus Faculty of Medicine, Damascus, Damascus Governorate, Syria, damascusuniversity.edu.sy; ^2^ Department of Internal Medicine, University of Damascus Faculty of Medicine, Damascus, Damascus Governorate, Syria, damascusuniversity.edu.sy; ^3^ Department of Histopathology, University of Damascus Faculty of Medicine, Damascus, Damascus Governorate, Syria, damascusuniversity.edu.sy

**Keywords:** benign ovarian tumors, case report, giant ovarian cyst, ovarian neoplasm, seromucinous cystadenoma

## Abstract

Giant ovarian tumors (GOTs) are uncommon ovary neoplasms with no universally accepted definition. They are typically noncancerous, with a mucinous subtype being the most common characteristic. Epithelial ovarian tumors are classified by cell type into serous, endometrioid, clear cell, mucinous, seromucinous, or transitional. Seromucinous cystadenomas are largely asymptomatic and can present with massive dimensions in late diagnoses, especially in low‐to‐middle‐income regions. In this case, a 26‐year‐old nulliparous Arab woman presented with a giant ovarian cyst in her reproductive age, undiagnosed for 9 years, which turned out to be a benign histopathology of seromucinous cystadenoma. The clinical presentation was nonspecific, as large ovarian cysts are often asymptomatic until they reach a substantial size to affect the quality of life and fertility. She had progressive abdominal swelling first noticed 6 years ago, but she started developing a feeling of fullness, constipation, nausea, vomiting, and urinary hesitancy with painful micturition 5 months prior to presentation with marked distress. The cystadenoma was excised without complications, through the exploratory laparotomy, and a left salpingo‐oophorectomy with right cystectomy was performed. Seromucinous cystadenomas were first classified in 2014 by the World Health Organization (WHO) as a new entity and a rare subtype of ovarian epithelial tumors. Patients with benign seromucinous cystadenomas have an excellent prognosis, especially with complete surgical removal. Raising awareness and increasing access to gynecological care in low‐ and middle‐income countries are essential for the long‐term well‐being and health of patients with giant ovarian cysts.

## 1. Introduction

Giant ovarian tumors (GOTs) are uncommon ovary neoplasms for which there is currently no universally accepted definition. Some researchers have characterized GOTs as tumors exceeding 10 cm in diameter, whereas others have established the threshold at 20 cm [1, 2]. Epithelial tumors are the most widespread among ovarian neoplasms [3]. It was reported that large cysts are typically predominantly noncancerous, such as the mucinous subtype (approximately 75% benign, 10% borderline, and 15% malignant) constituting approximately 15% of ovarian neoplasms [4].

The 2014 World Health Organization (WHO) Classification of Tumours of the Female Reproductive Organs introduced a new category of seromucinous neoplasms. This category consists of malignant (seromucinous carcinoma), borderline (seromucinous borderline tumor), and benign (seromucinous cystadenoma/adenofibroma) tumors [5]. The borderline category is the most comprehensively reported, but there was little published literature on benign seromucinous neoplasms until their inclusion in the WHO 2014 classification [6–8].

Giant cystadenomas are largely asymptomatic and can present with massive dimensions in late diagnoses, especially in low‐to‐middle‐income regions [1]. They present with gradual distension of the abdomen, nonspecific diffuse abdominal pain, and symptoms associated with organ compression [4]. We present here a case of a giant ovarian cyst undiagnosed for 9 years in a female of reproductive age, which turned out to be a benign pathology of seromucinous multiloculated cystadenoma.

## 2. Clinical Presentation

A 26‐year‐old nulliparous Arab woman, married for over 9 years, presented to the gynecology clinic at the Obstetrics and Gynecology University Hospital in Damascus, Syria. The patient was a nonsmoker and did not drink alcohol. She complained of persistent, dull, moderate, and generalized abdominal pain, most prominent in the hypogastrium. She also had a feeling of fullness, constipation, nausea, vomiting, and urinary hesitancy with painful micturition, starting 5 months prior to presentation. Furthermore, she had progressive abdominal swelling first noticed 6 years ago, in addition to menstrual irregularities persisting for more than 7 years.

**Figure 1:**
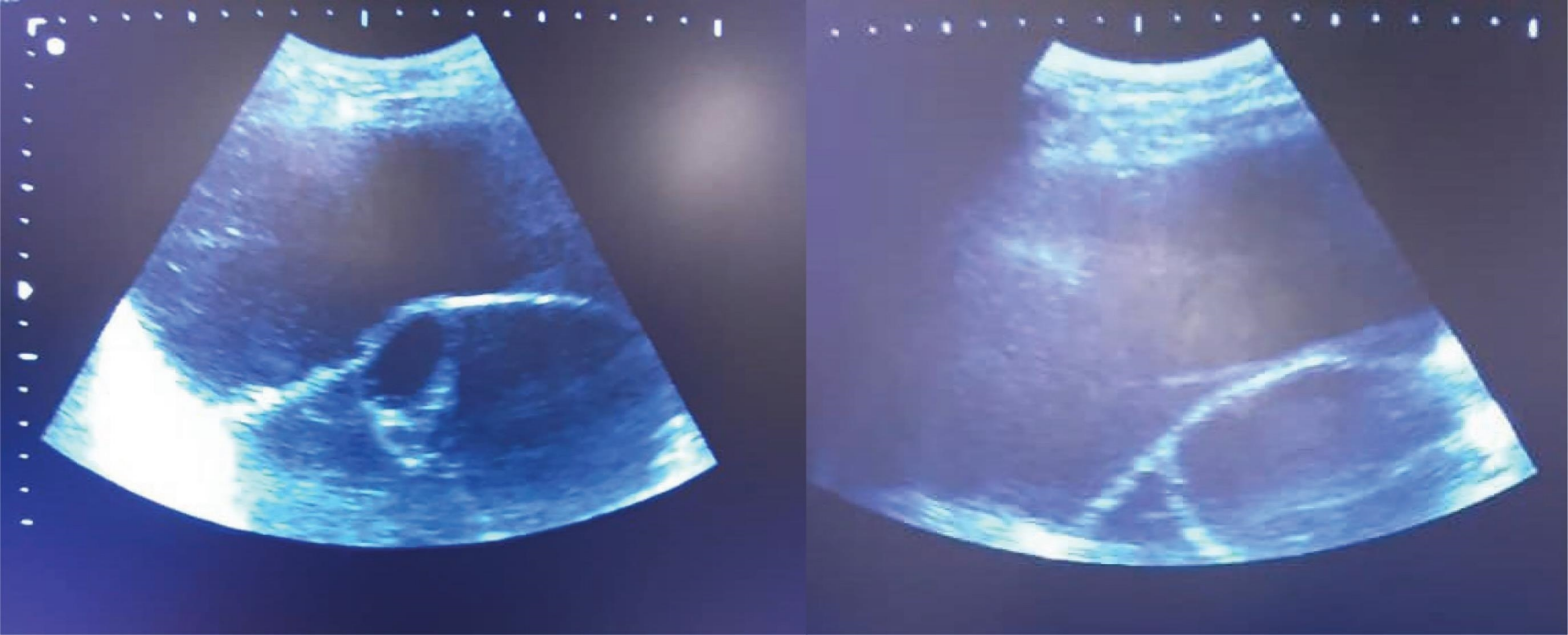
Ultrasonography views showing a multilocular cystic lesion with hyperechoic thin septations without solid components.

Her medical history revealed no other gynecological disorders except for a 12 cm ovarian cyst diagnosed 7 years ago, for which the patient stated that it shrank to 7 cm on follow‐up, but the patient stopped following up afterward. The rest of her family, medication, and medical history were unremarkable. Physical examination revealed normal vital signs, but the patient was in distress and generally unwell. On presentation, when asking the patient about the previous ovarian cyst symptoms, she admitted to noticing progressive abdominal enlargement and a generalized feeling of fullness, which, due to low health literacy, she attributed to gastrointestinal issues not warranting any checkup. The patient had a grossly distended, nonsoft, and tender abdomen, comparable to a pregnant female of 32 weeks. On deep palpation, there was a spherical mass, freely mobile, with a smooth surface and cystic consistency. No enlarged regional lymph nodes or organomegaly were identified. The patient was of low socioeconomic and educational status, which could have led her to postpone seeking medical evaluation, despite the grossly visible distended abdomen, which led to the case remaining undiagnosed for years. An abdominopelvic ultrasound scan showed a huge, thin‐walled, multiloculated cyst with septations and echogenically turbid fluid, extending from the pelvis to the epigastrium. No solid components or distinct papillary projections were observed originating from the cyst wall, as shown in Figure 1.

**Figure 2:**
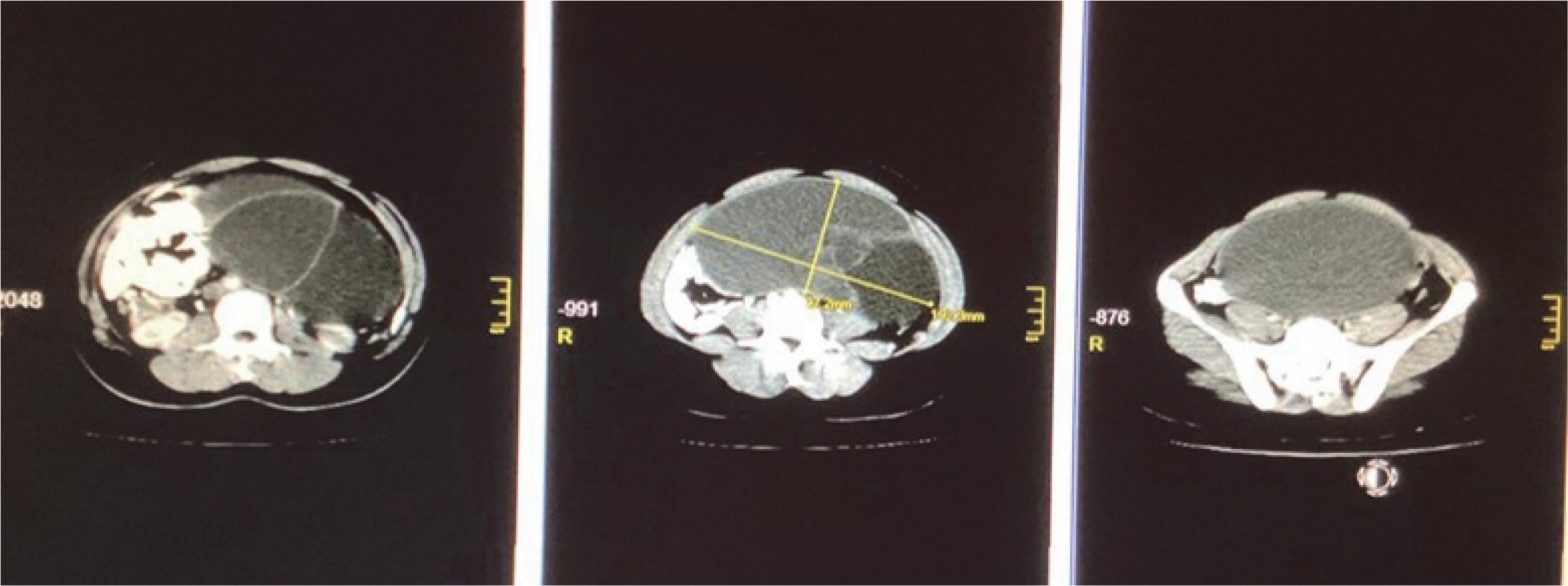
Computed tomography scan with contrast showing a huge cystic mass arising from the left adnexa.

A computed tomography scan with contrast was done (Figure 2), which found a huge cystic mass arising from the left adnexa, measuring 20.8 cm × 19.2 cm × 9.7 cm, with thin septation of 1 mm thickness, and slight contrast enhancement, but without calcification. It contained a fluid of homogenous echogenicity, without nodules or projections from the capsule. The cyst occupied nearly the entire pelvi‐abdominal cavity. No abnormally enlarged lymph nodes were noted in the periaortic and retroperitoneal spaces. Laboratory results demonstrated hemoglobin of 13.2 g/dL, white blood cells of 7.73 × 10^9^ cell/L, cancer antigen 125 (CA‐125) level was 17.3 U/mL, and her kidney and liver profile, glucose, urea, electrolytes, and urinalysis were all within normal ranges. Considering the large cystic mass, an exploratory laparotomy was performed under general anesthesia, carried out in a supine position through a midline incision.

**Figure 3:**
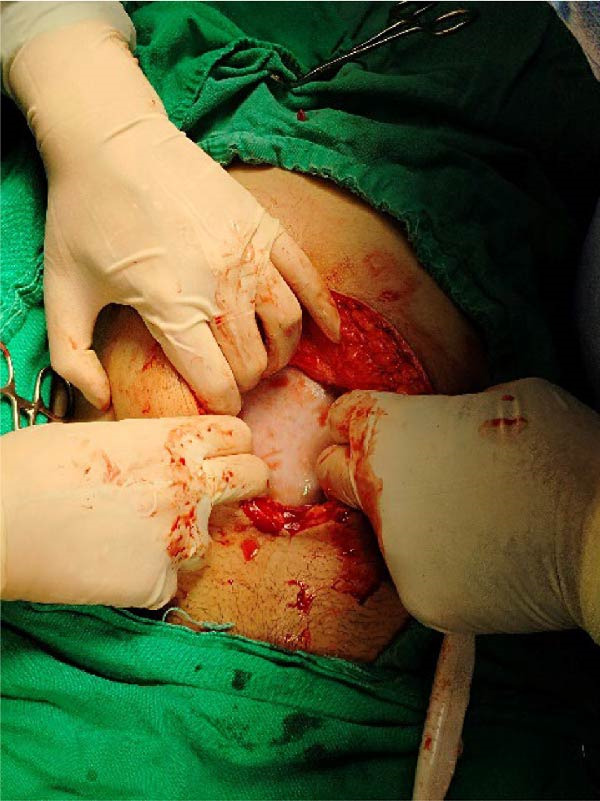
Gross view during laparotomy showing the shimmering surface of the cystic lesion.

**Figure 4:**
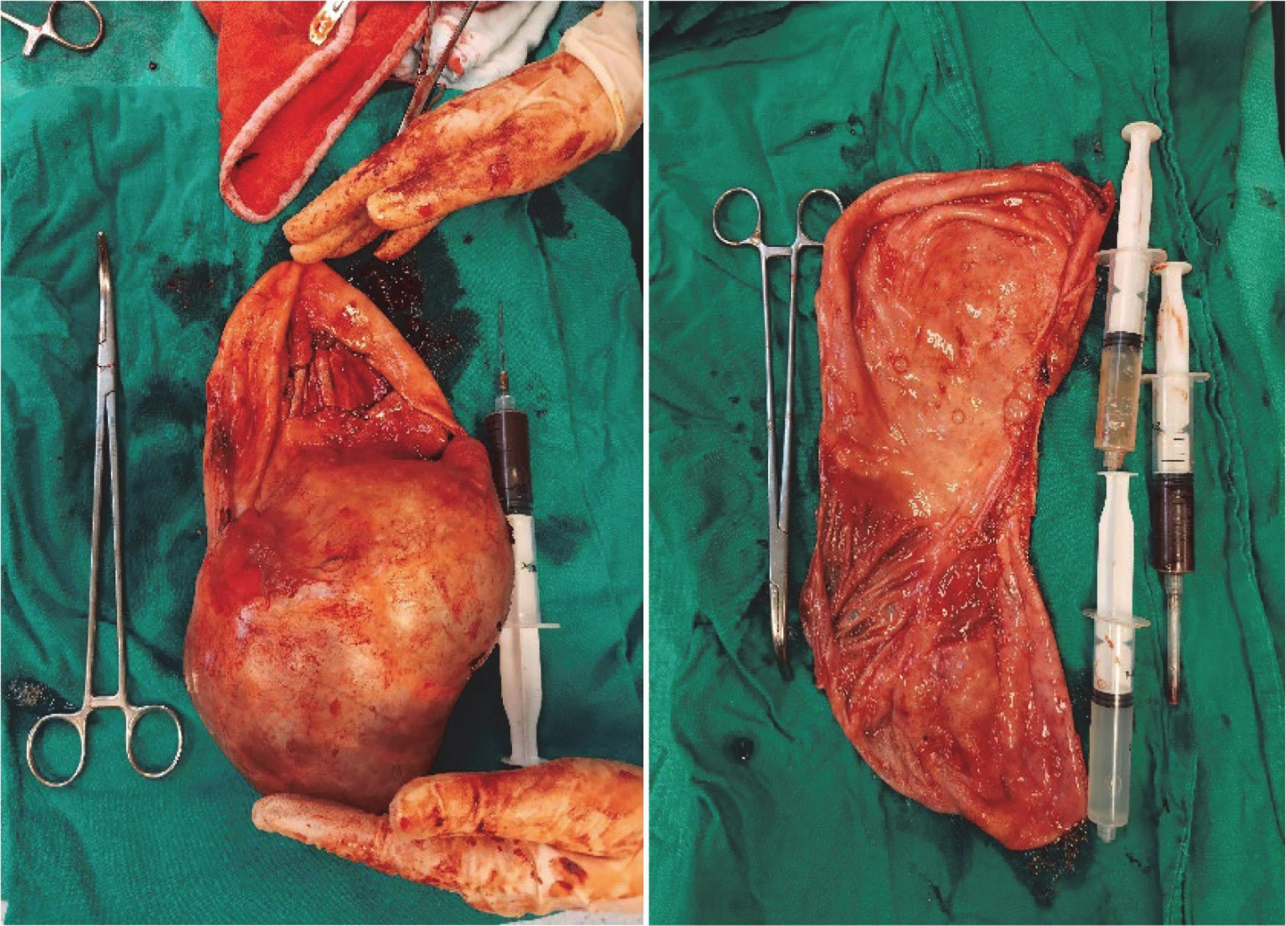
Gross examination of the cyst showing the multilocularity of the cyst, the internal surface, and three different samples drawn from different locules.

A large left adnexal cyst was found with a shimmering white surface, shown in Figure 3. On gross examination, the cyst was found to be multiloculated. The internal lining of the cyst wall was smooth, lacking any solid elements or papillary projections. The fluid aspirated from the upper locule appeared to be hemorrhagic, while the fluid in the other loculi was yellowish, with an estimated total of 2 L drained for gross inspection. Frozen section was not carried out, but the gross examination is shown in Figure 4. Additionally, the right fallopian tube and uterus were normal‐looking, but a 3 cm cystic mass of the right ovary was observed. A small amount of peritoneal fluid was also present, which was drained for examination. A left salpingo‐oophorectomy and right cystectomy were also performed, but no apparent lymph node involvement was noted.

Cytological examination of fluid aspirated from the cyst showed benign mucous cells, while one sample showed inflammatory cells, but no malignant cells were identified. The right cyst was histologically identified to be a corpus luteum cyst. No malignant cells were observed in the peritoneal fluid. Histopathological examination revealed the cyst to be partly consisting of a single layer of flattened to cuboidal lining, and also partly lined by columnar nonciliated epithelium with abundant mucin, all consistent with seromucinous cystadenoma of the left ovary, as shown in Figure 5. The patient was discharged from the hospital in good condition after 2 days since the complete surgical removal of the benign seromucinous cystadenoma was successful and uncomplicated. She was scheduled for long‐term follow‐up to identify any signs of recurrence or problems, especially that she had a history of recurrent ovarian cysts. Follow‐up after 2 months was within normal limits, and the patient reported relief from all the reported distressing symptoms. She was also referred to the fertility clinic for assessment of her infertility and further follow‐up and evaluation.

Figure 5(A) Serous component of epithelia of the cyst wall showing cuboidal cells typical for serous adenomatous component. (B) Mucinous component of epithelia of the cyst wall demonstrating columnar cells typical for mucin‐producing epithelium.

**Figure:**
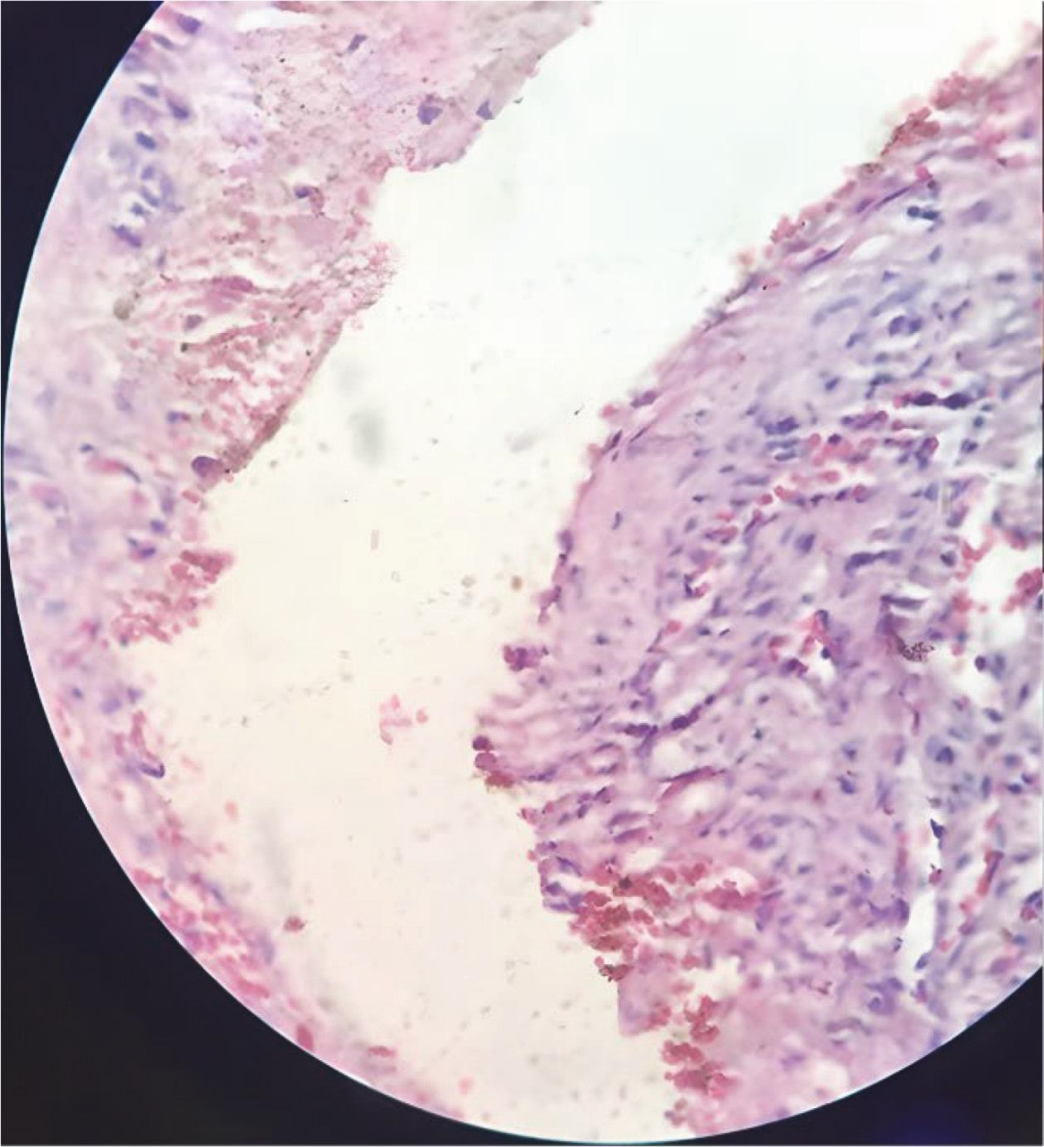
(A)

**Figure:**
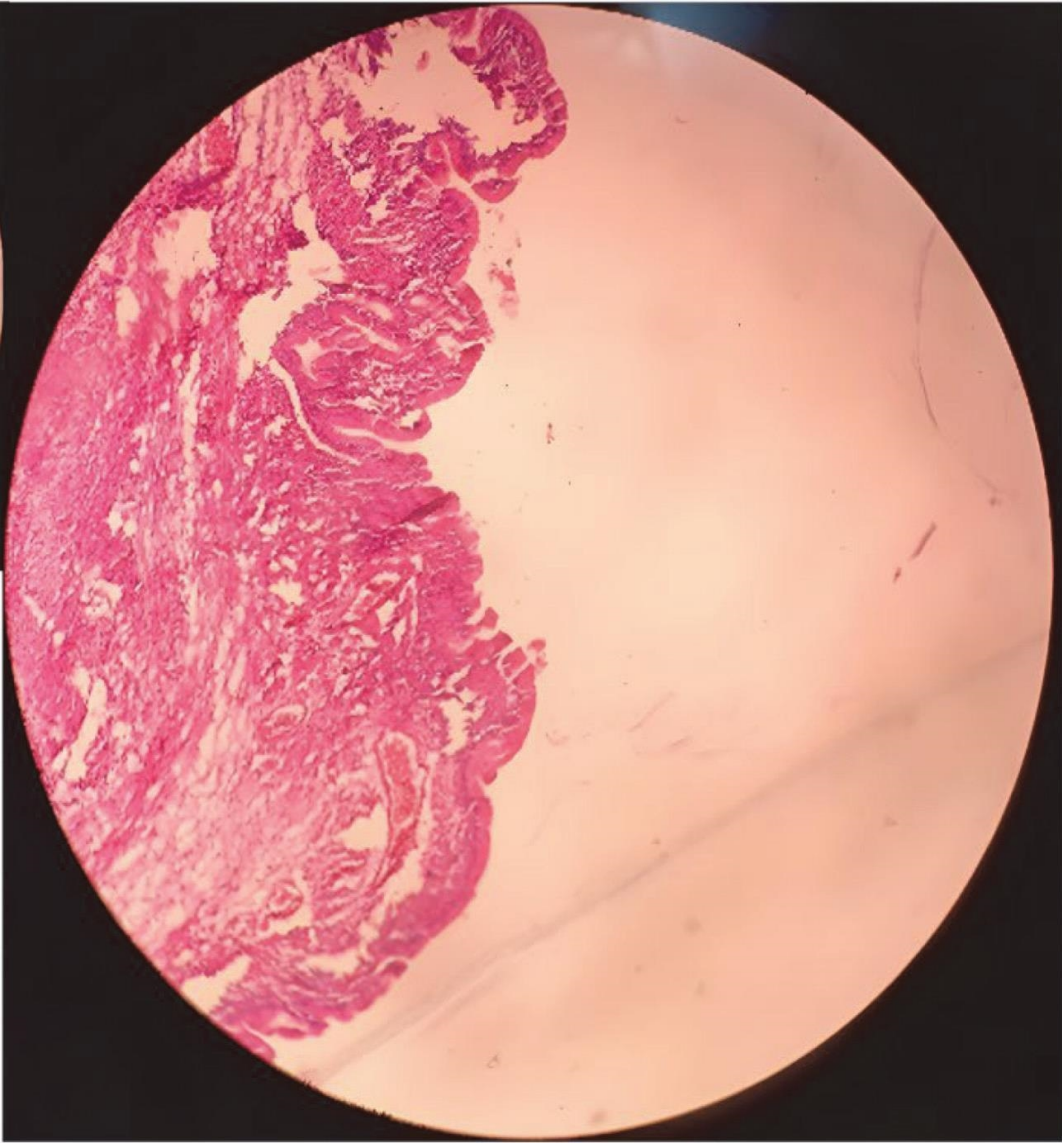
(B)

## 3. Discussion

Although ovarian cysts are a common finding in women during their reproductive age, and in females undergoing workups for infertility, they can develop into GOTs, which represent a diagnostic challenge. This is especially apparent in our low‐resource countries, where timely imaging and specialized care might be difficult to access.

The definition of GOTs varies in the literature; some studies have regarded them as larger than 20 cm in diameter, as in one previously by Khan et al. [9], while others have defined them as 10 cm or above in diameter. The cases of giant ovarian seromucinous tumors reported in the literature are outline in Table 1. In our patient, she had a giant ovarian cyst measuring 20.8 cm × 19.2 cm × 9.7 cm, which corresponded to both the aforementioned definitions of GOT. Knowing that large ovarian cysts are often asymptomatic until they reach a substantial size to affect the quality of life and fertility, the clinical presentation was nonspecific in our case as well [1, 2].

**Table 1:** Overview of reported giant seromucinous tumors in medical literature.

Reference	Location	Patient age	Tumor size/weight	Diagnosis (histopathology)	Clinical presentation	Management/surgical approach
Khan et al. [9]	India	48	28 cm × 20 cm × 15 cm; 12 kg; 10 L fluid	Benign serous cystadenoma	Chronic constipation (1 year), abdominal distension, vague discomfort; misdiagnosed as mesenteric cyst	Exploratory laparotomy; cyst excision with preservation of ovary
Murtani et al. [10]	Indonesia	48	51 cm × 40 cm × 19 cm	Seromucinous adenofibroma	10‐year history; anemia; hydronephrosis	Laparotomy + unilateral SO + debulking
Chen et al. [11]	China	65	30 cm × 22.8 cm × 12.4 cm	Seromucinous cystadenoma	7‐month distension; postmesenteric location	Single‐port laparoscopy (SPLS)
Sunanda et al. [12]	India	65	40 cm × 37 cm × 30 cm	Mixed serous and mucinous cystadenoma	4‐year history; “36‐week pregnancy” size	Laparotomy (TAH + BSO)
Fulop et al. [13]	Romania	49	27 cm × 26 cm × 12 cm	Seromucinous cystadenofibroma	Mimicked massive ascites	Laparotomy
Rodrigues‐Martins et al. [14]	Portugal	23	27 cm × 20 cm × 11 cm	Seromucinous cystadenoma (+ dermoid)	Fertility preservation requested	Bilateral cystectomy
Idrees et al. (case 4) [15]	Pakistan	50	29 cm × 25 cm	Seromucinous borderline tumor	Abdominal pain and swelling	Hysterectomy + BSO
Idrees et al. (case 6) [15]	Pakistan	34	23 cm × 13.5 cm	Seromucinous borderline tumor	Incidental/pain	Hysterectomy + BSO
Dinesh et al. [16]	India	Case series	Up to 20 cm (noted range)	Seromucinous adenofibroma	Varied; often associated with endometriosis (30%–70%)	Surgical removal (cystectomy or oophorectomy)

Abbreviations: SO, salpingo‐oophorectomy; SPLS, single‐port laparoscopic surgery; TAH + BSO, total abdominal hysterectomy + bilateral salpingo‐oophorectomy.

Diagnostic imaging, including abdominal ultrasound and computed tomography scans, is reported to be the cornerstone of planning for surgical interventions and the characterization of giant ovarian cysts [5, 17, 18]. Imaging studies in our case included abdominal ultrasonography and a CT scan, which showed benign features. In turn, such an absence of malignant features on imaging, together with normal CA‐125 levels, was clinically reassuring. However, histopathological examination was carried out in the end due to other prominent indications for surgical intervention, as is the case in most of the reported giant ovarian cysts. In our case, an exploratory laparotomy with a left salpingo‐oophorectomy and right cystectomy was done without any postoperative complications. Our patient had no compelling emergency indication for laparotomy, in accordance with Grigore et al.’s review on GOTs, in which the authors stated that only 12% had a compelling emergency for surgical intervention. Correspondingly, the management of giant ovarian cysts generally involves surgical intervention due to symptomatic presentations. The majority of septate cyst GOTs were also reported to be of a benign nature in comparison with cysts having solid components, where 18% of septate cysts turned out to be benign. Our seromucinous cystadenoma GOT had septations on ultrasonography and gross examination, and in turn, was of benign histopathology [19].

Epithelial ovarian tumors are classified by cell type into serous, endometrioid, clear cell, mucinous, seromucinous, or transitional. Seromucinous cystadenomas were first classified in 2014 by the WHO as a new entity and a rare subtype of ovarian epithelial tumors [5]. The inclusion of this novel category in the WHO classification has enabled us to understand these rare benign or malignant ovarian neoplasms better. Though there is little knowledge about the pathogenesis of seromucinous cystadenomas. Patients with benign seromucinous cystadenomas have an excellent prognosis, especially with complete surgical removal. They are considered to be derived from the Müllerian epithelium, and a paper by Kurman and Shih [20] published in 2016 reported that seromucinous tumors are of mullerian origin, and they have a close relation to endometriosis similar to the clear cell and endometrioid tumors.

In our case, the socioeconomic status of the patient also seemed to have had an influence on the delay in presenting for medical care despite all the symptoms of which she was suffering. Our patient had complete relief of symptoms, strongly suggesting the success and good prognosis of surgical management. Her infertility still required further evaluation, as an anatomical cause might not have been the only causation. This case emphasizes raising awareness and increasing access to gynecological care in low‐ and middle‐income countries. In addition, regular surveillance is also essential for these patients’ long‐term well‐being and health. Further research is required to understand the pathophysiology and determination of best management.

## 4. Conclusion

We report an unusual case of giant ovarian seromucinous cystadenoma in a 26‐year‐old female presenting late in her long history of infertility, and suffering from prominent abdominal compressive symptoms in spite of nonsuspicious imaging and CA‐125 level. Open laparotomy (left salpingo‐oophorectomy and right cystectomy) was performed for complete surgical removal. Postoperative recovery and disappearance of symptoms were prompt, illustrating the benignity of seromucinous tumors and their excellent prognosis if completely excised. Low socioeconomic status, and in turn inadequate women’s health literacy, which can be rectified through community health education programs, plays a vital role in educating women on such complaints being possibly due to gynecologic issues and to be taken seriously, further warranting checkup rather than mere dismissal as transient GI problems. Prompt identification and access to gynecologic care in limited resource settings are essential in order to prevent extensive morbidity, preserve reproduction and general women’s health in such settings.

## Author Contributions

Marah Kakeh and Mohammad Alhalabi analyzed and interpreted the patient data and drafted the manuscript. Wael Wazan and Rasha Abbassi contributed to gathering patient information and workup. Abeer Kanjawi performed histopathological examination. Haitham Abbassi reviewed and corrected the final manuscript.

## Funding

The authors declare that no funding was received for performing this study.

## Disclosure

All authors read and approved the final manuscript.

## Ethics Statement

All the procedures were approved by the Ethics Committee of Faculty of Medicine of Damascus University.

## Consent

Written informed consent was obtained from the patient for publication of this care report and any accompanying images. A copy of the written consent is available for review by the Editor‐in‐Chief of this journal.

## Conflicts of Interest

The authors declare no conflicts of interest.

## Supporting Information

Additional supporting information can be found online in the Supporting Information section.

## Supporting information


**Supporting Information** A populated CARE checklist according to the CARE guidelines for clinical case reporting [21] is provided along with the manuscript, and can be accessed in the relevant section online.

## Data Availability

Data sharing is not applicable to this article as no datasets were generated or analyzed during the current study.
