# Supplementary material for: A Rare Massive Seromucinous Cystadenoma of the Ovary in a 26‐Year‐Old Female Persisting for 9 Years: A Case Report
Source: Case Rep Obstet Gynecol. 2026 Apr 9;2026:5887168. doi: 10.1155/crog/5887168 (PMC13066506; doi:10.1155/crog/5887168)
Supplement: Supplementary file 1 — Supporting Information A populated CARE checklist according to the CARE guidelines for clinical case reporting [21] is provided along with the manuscript, and can be accessed in the relevant section online. [file CROG-2026-5887168-s001.pdf]

CARE
case report guidelines

CARE Checklist of information to include when writing a case report

(cc) BY-NC-ND

Topic	Item	Checklist item description	Reported on Line
Title	1	The diagnosis or intervention of primary focus followed by the words “case report”	1
Key Words	2	2 to 5 key words that identify diagnoses or interventions in this case report, including "case report"	45
Abstract (no references)	3a	Introduction: What is unique about this case and what does it add to the scientific literature?	31
	3b	Main symptoms and/or important clinical findings	35
	3c	The main diagnoses, therapeutic interventions, and outcomes	33 & 38
	3d	Conclusion—What is the main “take-away” lesson(s) from this case?	43
Introduction	4	One or two paragraphs summarizing why this case is unique (**may include references**)	49
Patient Information	5a	De-identified patient specific information.	70
	5b	Primary concerns and symptoms of the patient.	72
	5c	Medical, family, and psycho-social history including relevant genetic information	77
	5d	Relevant past interventions with outcomes	77
Clinical Findings	6	Describe significant physical examination (PE) and important clinical findings.	80
Timeline	7	Historical and current information from this episode of care organized as a timeline	75
Diagnostic Assessment	8a	Diagnostic testing (such as PE, laboratory testing, imaging, surveys).	90 & 94
	8b	Diagnostic challenges (such as access to testing, financial, or cultural)	87
	8c	Diagnosis (including other diagnoses considered)	117
	8d	Prognosis (such as staging in oncology) where applicable	121
Therapeutic Intervention	9a	Types of therapeutic intervention (such as pharmacologic, surgical, preventive, self-care)	102
	9b	Administration of therapeutic intervention (such as dosage, strength, duration)	N/A
	9c	Changes in therapeutic intervention (with rationale)	N/A
Follow-up and Outcomes	10a	Clinician and patient-assessed outcomes (if available)	120
	10b	Important follow-up diagnostic and other test results	122
	10c	Intervention adherence and tolerability (How was this assessed?)	N/A
	10d	Adverse and unanticipated events	N/A
Discussion	11a	A scientific discussion of the strengths AND limitations associated with this case report	227
	11b	Discussion of the relevant medical literature **with references**.	189 & 209
	11c	The scientific rationale for any conclusions (including assessment of possible causes)	223
	11d	The primary “take-away” lessons of this case report (without references) in a one paragraph conclusion	236
Patient Perspective	12	The patient should share their perspective in one to two paragraphs on the treatment(s) they received	228
Informed Consent	13	Did the patient give informed consent? Please provide if requested	Yes ☑ No ☐
